# Nitrogen Deposition Amplifies the Legacy Effects of Plant Invasion

**DOI:** 10.3390/plants13010072

**Published:** 2023-12-25

**Authors:** Miaomiao Cui, Haochen Yu, Xue Fan, Mohsin Nawaz, Junjie Lian, Shihong Liu, Zhaoqi Zhu, Haiyan Zhang, Daolin Du, Guangqian Ren

**Affiliations:** 1 School of the Environment and Safety Engineering, Jiangsu University, Zhenjiang 212013, Chinamohsinnawaz2676@gmail.com (M.N.); zzq2292023@163.com (Z.Z.); 2 School of Inspection and Testing Certification, Changzhou Vocational Institute of Engineering, Changzhou 213164, China; 3 Jingjiang College, Jiangsu University, Zhenjiang 212013, China; 4 Institute of Environment and Ecology, Jiangsu University, Zhenjiang 212013, China; 5 School of Emergency Management, Jiangsu University, Zhenjiang 212013, China; 6 Jiangsu Province Engineering Research Center of Green Technology and Contingency Management for Emerging Pollutants, Jiangsu University, Zhenjiang 212013, China; 7 Jiangsu Collaborative Innovation Center of Technology and Material of Water Treatment, Suzhou University of Science and Technology, Suzhou 215009, China

**Keywords:** plant invasion, population dynamics, nitrogen deposition, legacy effects, secondary invasion

## Abstract

The legacy effects of invasive plant species can hinder the recovery of native communities, especially under nitrogen deposition conditions, where invasive species show growth advantages and trigger secondary invasions in controlled areas. Therefore, it is crucial to thoroughly investigate the effects of nitrogen deposition on the legacy effects of plant invasions and their mechanisms. The hypotheses of this study are as follows: (1) Nitrogen deposition amplifies the legacy effects of plant invasion. This phenomenon was investigated by analysing four potential mechanisms covering community system structure, nitrogen metabolism, geochemical cycles, and microbial mechanisms. The results suggest that microorganisms drive plant–soil feedback processes, even regulating or limiting other factors. (2) The impact of nitrogen deposition on the legacy effects of plant invasions may be intensified primarily through enhanced nitrogen metabolism via microbial anaerobes bacteria. Essential insights into invasion ecology and ecological management have been provided by analysing how nitrogen-fixing bacteria improve nitrogen metabolism and establish sustainable methods for controlling invasive plant species. This in-depth study contributes to our better understanding of the lasting effects of plant invasions on ecosystems and provides valuable guidance for future ecological management.

## 1. Introduction

Biological invasion is a global phenomenon driven by the increasingly interconnected world, rapid human population growth, and drastic changes in the global environment [1,2,3]. Invasive species can invade the local environment of multiple types and plant species in various ways, causing continuous and frequent severe disturbances and hazards to regional and global ecology, the environment, and human activities [4]. Yet, as we grapple with the repercussions of this phenomenon, it becomes imperative to delve into the intricate dynamics between two categories of plants: weeds and invasive plants. Weeds are often synonymous with invasive plants, but invasive plants and weeds are two concepts that, although somewhat related, are distinct in definition and impact. Invasive plants refer to non-native or exotic plants. They usually have strong competitiveness and phenotypic plasticity [5,6], can actively respond to changes in the external environment (Table 1), and rapidly spread and establish populations in new ecosystems, causing native plants to be replaced or even disappear. Invasive plants can change soil properties and destroy habitats, causing huge impacts on ecosystems and biodiversity. Weeds are undesirable plants that occur in artificial or agricultural environments. Weeds are often closely associated with human activities and land use, and they may compete for the nutritional resources of crops. While weeds do not necessarily become invasive plants, their overgrowth in certain environments can cause a range of agricultural problems. Therefore, invasive plants are a type of weed that comes from outside the area and poses a threat to the local ecosystem, while weeds are a part of the local ecosystem and biodiversity. In the practical activities of ecological protection and agricultural management, the ecological characteristics of plants should be comprehensively considered, and effective measures should be taken to protect the stability of the ecosystem and the sustainability of agriculture.

Approximately 17% of the Earth’s land surface is highly invaded, and it is predicted that the number of non-native vascular plants in temperate Asia will increase by 41% by 2050 [12]. Consequently, the current focus has shifted from safeguarding native species to managing alien species to protect and restore native biodiversity and ecosystem services [13]. While numerous studies have examined the direct impacts of invasive species, it is essential to recognise that their legacy effects continue to impact ecosystems long after they are removed. Understanding and addressing 

**Figure 1:**
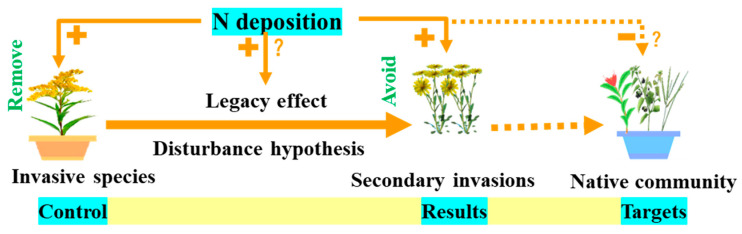
Legacy effect of nitrogen deposition enhancing plant invasion (solid line is the research focus, dotted line is the final goal, “+” is the positive effect, “−” is the negative effect, and “?” indicates the problems to be solved).

Managing invasive plants in natural ecosystems is complex, and efforts to restore native plant communities and their ecosystem functions often fail. These attempts may even lead to more severe secondary invasions (Figure 1). Secondary invasions occur when controlling the targeted invasive species results in non-target invasive plants proliferating, forming new invasive species with physical, chemical, biological, or other control measures. However, secondary invasions were quantified in only 29% of invasion management studies, but of those, 89% of secondary intrusions were harmful [14]. As a result, secondary invasions prolong and intensify the threats invasive plants pose to ecosystems and challenge invasive plants and land management practices. The essence of such problems is an unclear understanding of legacy effects and their mechanisms (Figure 1).

The legacy effect, initially introduced in ecology during the early 1990s, pertains to the removal, disappearance, or cessation of one or more species or environmental elements. Its effects on ecosystem components, linkages, structures, functions, and dynamics may last considerably longer [15]. Previous studies have revealed that the impact of plant invasion on ecosystems has apparent legacy effects that persist even after removing invasive plant species. It should be highlighted that the legacy effects of plant invasion begin with the control of invasive plants and with the engineering effects produced during their growth phase. The legacy effects also seem to be closely related to invasion history. Peterson et al. [13] demonstrated that the coverage rate of targeted invasive plants decreased by over 80% following biological control and herbicide treatment. However, the number of exotic non-target monocotyledon plants increased almost eight times. Nsikani et al. [16] found that while removing targeted invasive plants resulted in the re-establishment of a large part of the ecological population, it still primarily constituted a secondary invasive plant population. More than 50% of studies show that after measures to control target invasive species are implemented, non-target alien plants increase rapidly and cause serious harm to the ecosystem [17]. The question arises: why are more managed areas experiencing secondary invasions?

Legacy effects are vital regulatory pathways in ecosystem relationship networks, significantly impacting the interplay between plants and biological and abiotic environmental factors [18]. Previous research has indicated that secondary invasions may be significantly influenced by legacy effects, which may be attributed to (1) empty ecological niches resulting from the removal of targeted invasive species, (2) alterations in soil nutrient cycling due to litter decomposition, and (3) the impact of root exudates on the structure of the soil microbial community. Additionally, other legacy effects, including inter-species interactions, nutrient competition, nitrogen metabolism, and seed bank insurance, could also act as factors influencing secondary invasions [16]. These factors partly explain the secondary invasion phenomenon but require more comprehensiveness and systematic analysis. In particular, the global climate and environment have undergone drastic changes in recent years, increasing the frequency of secondary invasions [19]. As a result, it is crucial to conduct more in-depth and systematic research that combines legacy effects with environmental changes.

## 2. The Importance of Nitrogen Deposition in the Context of Invasive Plants and Implications for Legacy Effects

### 2.1. Importance of Nitrogen Deposition in the Context of Invasive Plants

Due to anthropogenic activities such as burning fossil fuels and applying inorganic fertilisers, environmental change factors like nitrogen deposition and climate warming have become more complex and substantial than initially expected [20], and this trend is expected to continue. China’s land-based ecosystem has received the highest global nitrogen residue levels, ranking among the top three worldwide [21]. Nitrogen, as an essential nutrient for plant growth, significantly increases the available nitrogen content of soil and alters the soil’s microbial structure. It also modifies the activity and concentration of enzymes associated with photosynthesis in leaves [22,23]. Numerous prior studies have demonstrated that nitrogen deposition can substantially boost the successful invasion of invasive plant species. Therefore, nitrogen deposition has legacy effects and will persist even after implementing invasive plant control measures [24]. Therefore, it is imperative to understand the impact of nitrogen deposition on the legacy effects of invasion and the associated mechanisms.

Fang et al. [25] demonstrated that environmental climate change is the primary factor influencing invasive plant spread and distribution. Because invasive plants have greater nutrient use efficiency, habitats high in nutrients are more susceptible to invasion by non-native plants [8]. Consequently, nitrogen deposition can facilitate plant invasion directly and/or indirectly by changing soil microbial communities’ physical and chemical properties [26]. Nevertheless, insufficient research examines the legacy effect of nitrogen deposition on plant invasion. Perring et al. [27] examined the characteristics of understory community abundance and plant height in Europe after 1800. The study found that the influence of global environmental changes, such as nitrogen deposition, on the composition of plant communities varied based on the legacy effects of historical land management. Elgersma et al. [28] demonstrated that the effectiveness of controlling invasive *Typha* depended on exogenous nitrogen input. These findings suggest that artificial invasive plant control legacy effects interact with natural environmental changes, potentially heightening the risk of secondary invasion (Figure 1).

### 2.2. The Mechanism of Nitrogen Deposition Affecting Legacy Effects

Effective management of invasive plant species is critical to maintaining healthy habitats. The disturbance hypothesis suggests that the disturbance can cause ecological niche vacancies and increase soil nutrient levels [29,30]. However, the nitrogen deposition increase will likely have a superimposed effect on the nutrient increase caused by controlling invasive plants. Such nutrient-rich habitats have higher invasiveness than poor ones. Invasive species encounter obstacles but can better cope with environmental changes (disturbance and nitrogen deposition) [31]. On the other hand, habitat disturbance caused by measures to control invasive plants may also harm native species to varying degrees. In an ecological legacy experiment, Nsikani et al. [32] discovered that the rise in soil nitrogen levels following the eradication of the invasive *Acacia saligna* significantly facilitated the development of the roots and branches of several secondary invasive plants. Hence, we propose the first point that nitrogen deposition amplifies the legacy effects of plant invasion. Although the roles of legacy effects and nitrogen in ecosystem change are well documented, their integration and interactions remain relatively unknown. This perspective addresses the under-explored interactions between legacy effects, nitrogen deposition, and secondary invasions. It further investigates the complex mechanisms by which nitrogen deposition amplifies the legacy effects of plant invasions and explores the challenges posed by legacy effects and increasing nitrogen deposition. 

**Figure 2:**
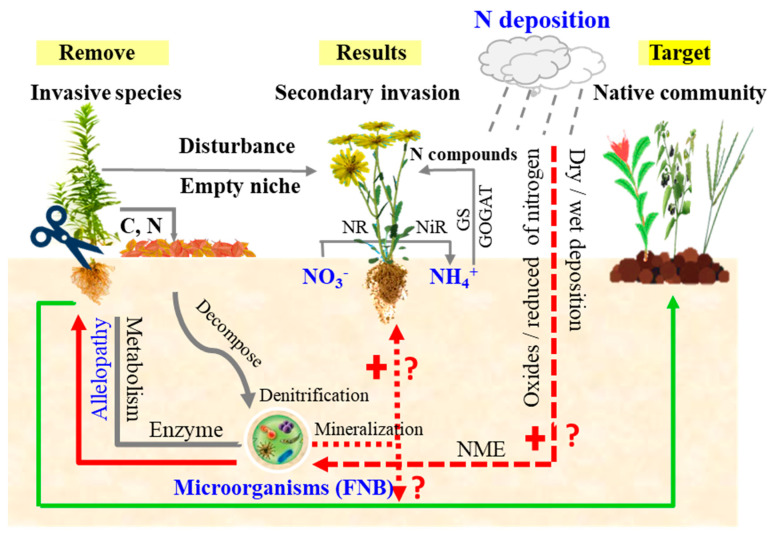
Process and mechanism of nitrogen deposition participating in the legacy effect of plant invasion (the solid line indicates existing evidence, the dotted line is the target to be achieved, “+” is the positive effect, “−” is the negative effect, and “?” indicates the problems to be solved).

(1) The community system structure level, characterized by interspecific competition, population dynamics, and ecological niche, is impacted by the rapid increase in nitrogen deposition. Nitrogen deposition can enhance the invasion of exotic plants by changing regional soil nutrient levels, thus reshaping the community structure. Seasonal nitrogen deposition leads to a notable rise in invasive plants′ biomass and root exudates, resulting in increased harm to native plants [33]. When target invasive plants are removed, the broad niche they occupy can lead to the competitively driven release and widespread proliferation of non-target invasive plants that were previously restricted (Figure 2). The prolonged presence of invasive plants in the area will likely establish a mutually constraining relationship with other species. Removing such plants could destabilise the ecosystem structure and disrupt interspecific competition, ultimately diminishing the community’s ability to resist invasion. In addition, the constant fluctuation of regional nutrient levels caused by nitrogen deposition elevates the potential for secondary invasions [34]. While overnutrition may arise in certain areas, investigations have indicated that invasive plants possess greater environmental adaptability than native plants.

(2) Nitrogen metabolism levels are characterised by the absorption and utilisation of nitrogen and enzyme activity in plants. This process is fundamental to plant physiology and is crucial in geochemical cycles. Nitrogen metabolism involves the absorption, utilisation, and enzyme activity related to nitrogen in plants, playing a fundamental role in plant physiology and geochemical cycles. Plant nitrogen assimilation converts inorganic nitrogen into organic nitrogen in plants, and plants can directly utilise ammonium nitrogen and nitrate nitrogen in the soil [35]. 

For nitrate nitrogen, plants require reduction to form NH_4_^+^, which is performed by nitrate reductase (NR) and nitrite reductase (NiR). Nitrogen donors such as glutamine and glutamic acid are then used to synthesise nucleotides, chlorophyll, and other compounds. This promotes the synthesis of proteins and other nitrogenous compounds [36]. Nitrogen assimilation in cells is also associated with aspartate synthetase (As) and aspartate aminotransferase (Asp AT). There are two aspects concerning nitrogen metabolism: soil’s fertiliser supply capacity and plants’ conversion capacity to nitrate nitrogen. Concerning metabolism, soil’s nitrogen level significantly correlates with the nitrate reductase, soil urease, and protease of invasive plants [37]. Therefore, during the growth stage of invasive plants, nitrogen deposition can impact legacy effects by altering the metabolic activity of both soil and plants. In the residual stage, available nitrogen levels may be elevated primarily through changes in soil enzyme activity, raising the potential for secondary invasion.

(3) Litter quality and mineralization rate are critical factors in geochemical cycling. Soil nitrogen predominantly exists in organic form, with a significant portion originating from litter and plant residues. Research indicates invasive plant litter contains a higher nitrogen content than native plants, providing favourable nutritional conditions for secondary invasion (Figure 2). For instance, *Chrysanthemoides monilifera* spp. *rotundata* exhibits a high degree of invasiveness in soils with varying nutrient levels and can enhance soil nutrients through litter decomposition in the area of its invasion. This presents a potential for secondary invasion by *Asparagus aethiopicus*, which only invades in high-nutrient soils [38]. Alien plant invasions increase ecosystem litter decomposition, increasing plant nitrogen content by 86–112% [39]. The increase in plant nitrogen content will further increase the nitrogen content of litter in the ecosystem (+38%). Litter decomposition and nutrient release were hastened, with a significant correlation observed with nitrification guided by ammonia-oxidizing archaea (AOA) [35]. Nitrogen deposition helps reduce an organism’s carbon-to-nitrogen ratio, increasing its mass and accelerating litter decomposition. It also creates favourable conditions for invading non-native plants.

(4) Microbial levels are characterised by mycorrhizal symbiosis and soil nitrogen-fixing bacteria. Plants affect the activities and structure of soil organisms through allelopathic substances, further affecting litter’s decomposition and nutrient release. Liu et al. [40] demonstrated that the invasive plant *Mikania micrantha* metabolites participate in the nitrogen cycle by increasing the abundance of microorganisms and improving nitrogen utilization. Elgersma et al. [41] discovered that the structure and function of microbial communities were primarily determined by the type of vegetation that existed two years ago rather than by current vegetation. After the successful invasion of non-native plants, soil nitrogen-fixing, and ammonia-oxidizing bacteria and fungi, soil nutrient content and related enzyme activities will increase, further exacerbating the spread of invasive plant species. Wang et al. [42] demonstrated that nitrogen deposition can increase the abundance of soil nitrogen-fixing bacteria (SNB) and may act in conjunction with invasive plants and promote SNB dominance. Regarding mycorrhizal interactions, the symbiotic relationship between invasive species and native microorganisms is more adaptable than between native species and native microorganisms, giving it a clear competitive advantage. In addition, the interaction between invasive species and microorganisms in the rhizosphere responds significantly more to nitrogen deposition than between native species and microorganisms. In particular, nitrogen deposition reduces the benefits of invasive plants from the mycorrhizal symbiosis system, allowing them to form a monopoly on nitrogen supply and reducing reliance on microorganisms to better adapt to the legacy effects of invasive plant control. 

## 3. Microorganisms Play a Critical Role in the Legacy Effects of Nitrogen Deposition on Invasive Plants

Essentially, the impact of nitrogen deposition on the legacy effects of plant invasion may be mainly through ecological mechanisms such as plant population dynamics, nitrogen metabolism pathways, soil nitrogen mineralization rates, and microbial functional bacteria. Further analysis has revealed that microorganisms are crucial in the plant–soil feedback process. As important driving factors, microorganisms are closely connected with plant community structure and soil geochemical cycles and even regulate or restrict the regular operation of other factors. First, microorganisms can influence plant population growth and competitiveness by providing nutrients to plants or through symbiotic relationships with plants. Secondly, they participate in the decomposition of organic matter and further transformation of nitrogen to regulate nitrogen availability in the soil. In addition, microorganisms can also promote the activity of their nitrogen metabolism pathways through symbiotic relationships with plants. In summary, microorganisms can influence the legacy effects of nitrogen deposition on invasive plants through multiple mechanisms. Ouyang et al. [43] demonstrated that exotic species with high nitrogen fixation ability had significantly higher nitrate reductase activity than native plants, and inoculation with *Azotobacter* significantly promoted nitrate reductase and glutamine synthetase activities in plant leaves [44,45]. Additionally, it significantly improved the activities of glutamate oxaloacetate transaminase and glutamic pyruvic transaminase in exotic species but had no significant impact on native species. Therefore, the second point proposes that (2) the exacerbation of nitrogen deposition on the legacy effects of plant invasion may primarily be achieved through enhanced nitrogen metabolism via microbial anaerobes.

Removing invasive plants is a widespread technique for reinstating indigenous ecosystems [46]. Our analyses show that the legacy effects of plant invasions can affect plant communities’ dynamic succession and ecological processes, leading to the emergence of secondary invasions. This means that even when invasive species are removed, their effects on the ecosystem persist, which has important practical implications for ecosystem restoration and management [47]. Second, nitrogen deposition is essential in regulating plant invasions’ legacy effects [48]. Nitrogen deposition can alter soil nitrogen availability, affecting plant invasions’ legacy effects, suggesting that global nitrogen deposition may further exacerbate the legacy effects of invasive species. An in-depth study of the legacy effects of plant invasions and their relationship with nitrogen deposition is essential for adopting a series of measures in practical ecological management to mitigate the negative impacts of the legacy effects. Among them, early management and monitoring of invasive plants are particularly urgent. Timely detection and removal of invasive species can effectively reduce their impact on the ecosystem, reducing legacy impacts’ negative effects. This management strategy covers multiple levels, including physical, chemical, and biological control methods [49]. Through early intervention and regular monitoring, we can hopefully minimise the disruption of invasive plants to ecosystem structure and function. Combining physical, chemical, and biological controls can lead to more comprehensive and sustainable ecological management in various situations. This approach not only preserves local biodiversity but also mitigates the further expansion of the legacy effects of nitrogen deposition on plant invasions, providing a more reliable guarantee of ecological balance. In addition, in the process of restoring vegetation and ecosystems, the impact of legacy effects on plant communities should be considered. This can improve ecosystem stability and resilience by introducing native plant species and increasing plant diversity [50]. At the same time, reasonable soil management and nutrient regulation can also reduce the role of legacy effects in promoting plant invasion. For example, through proper fertilisation and soil improvement measures, nitrogen supply in the soil can be regulated, and the competitive advantage of invasive species can be reduced [51].

This analysis relies primarily on the existing literature and available data, acknowledging possible incompleteness and limitations. Plant invasion and its legacy effects on the ecosystem are complex ecological phenomena also affected by geographical factors, climatic conditions, and human activities. Due to limitations in data availability, this analysis may not cover all possible factors affecting these processes. Furthermore, this analysis is primarily based on the current state of knowledge and technological capabilities, but the understanding of legacy impacts associated with plant invasions may evolve. Therefore, it is critical to recognise the dynamic nature of the field and anticipate potential changes in perspectives and insights. To better understand the impact and management of the legacy effects of plant invasions, future analyses will refine existing insights and address data gaps to fully understand other ecological processes that influence the legacy effects of invasive plants.

## 4. Conclusions

Analysing the legacy effects of plant invasions and nitrogen deposition can reveal their relationships and impacts on plant communities and ecosystems. This analysis found that the legacy effects of invasive plants can influence the occurrence of secondary invasions. Nitrogen deposition plays a critical role in the legacy effects of invasive plants among global environmental change drivers. The genetic effects of plant invasion in response to nitrogen deposition will be explained by studying the critical mechanisms by which microbial nitrogen-fixing bacteria promote nitrogen metabolism. Further research and applications are critical to protecting and restoring ecosystem health, managing invasive species’ impacts and legacy effects, and providing theoretical guidance for evolving ecological practice. At the same time, we recognise that ecological management should consider the comprehensive effects of multiple factors, such as biological legacies, environmental changes, and human management actions. Therefore, future research should further explore and understand the interaction of these factors to develop more comprehensive and effective invasive species management strategies to protect and restore ecosystem health.

## Figures and Tables

**Table 1:** Character analysis of weeds and invasive plants. The direction of the arrow indicates a stronger performance.

Invasive Species	Competition	Phenotype	Weed
*Chromolaena odorata*	←	→	*Urena lobata* [7]
*Solidago canadensis*	←	←	*Pterocypsela laciniata* [8]
*Galinsoga quadriradiata*	←	←	*Heteropappus hispidus* [9]
*Lepidium virginicum*	←	←	*Agropyron cristatum* [10]
*Wedelia trilobata*	←	←	*Wedelia chinensis* [11]

## Data Availability

All data generated or analysed during this study are included in this article.
